# Supplementary material for: Engineering Chimeras by Fusing Plant Receptor-like Kinase EMS1 and BRI1 Reveals the Two Receptors’ Structural Specificity and Molecular Mechanisms
Source: Int J Mol Sci. 2022 Feb 15;23(4):2155. doi: 10.3390/ijms23042155 (PMC8876890; doi:10.3390/ijms23042155)
Supplement: Supplementary file 1 [file ijms-23-02155-s001.zip › ijms-1590569-supplementary.pdf]

Engineering chimeras by fusing plant receptor-like kinase EMS1 and BRI1 reveals the two receptors structural specificity and molecular mechanisms

Qunwei Bai[1], Chenxi Li[1], Lei Wu[1], Huan liu[1], Hongyan Ren[1], Guishuang Li[1], Qiuling Wang[2], Guang Wu[1], Bowen Zheng[1,*]

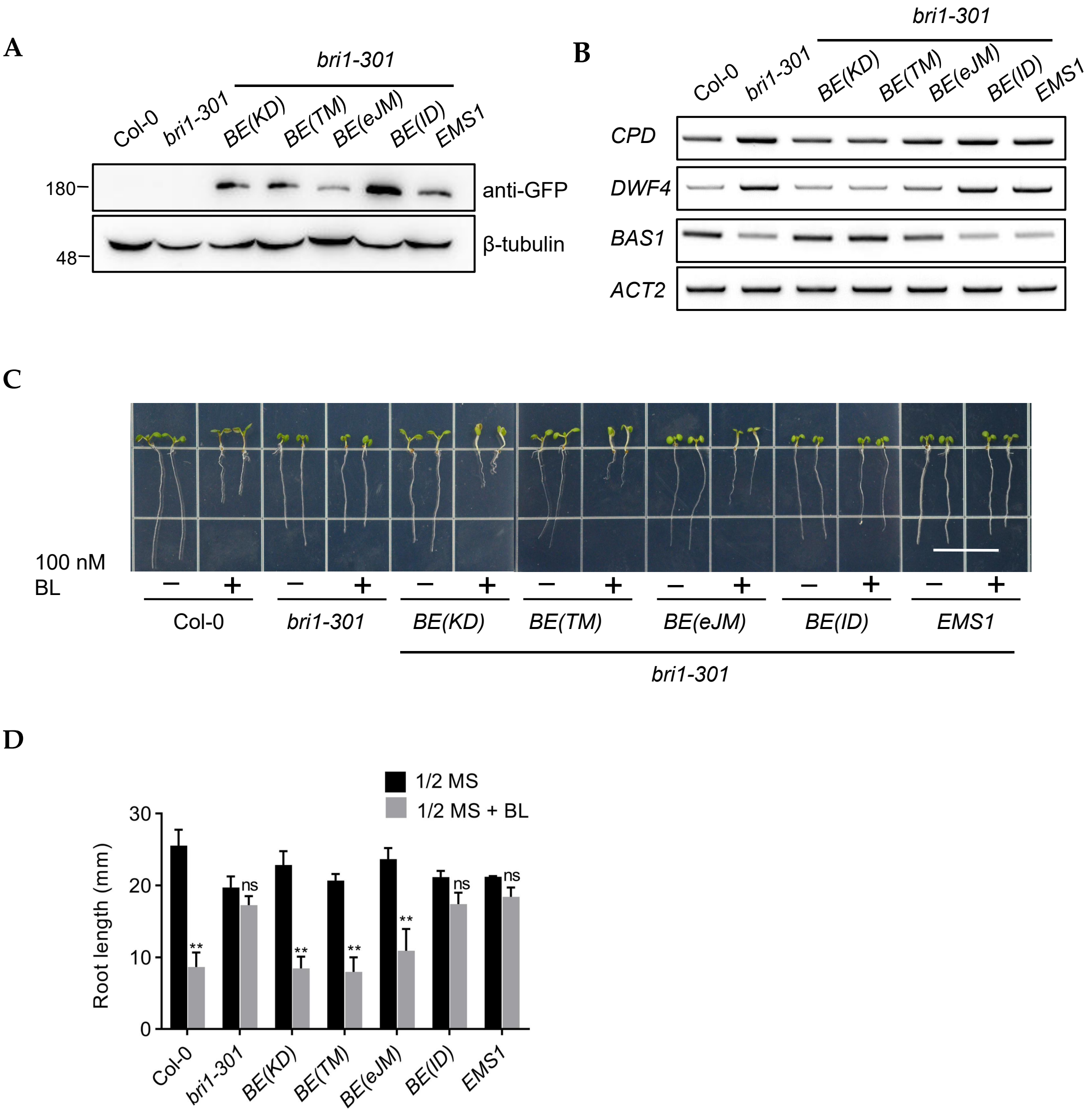

Figure S1. BE(KD), BE(TM) and BE(ID) could completely or partially recover *bri1-301*. (**A**) The protein expression levels of *BE(KD)/bri1-301, BE(TM)/bri1-301, BE(eJM)/bri1-301, BE(ID)/bri1-301* and *EMS1/bri1-301* transgenic plants. Col-0 and bri1-301 were used as the negative control. β-tubulin served as a loading control. (**B**) Semi-quantitative PCR analysis of BR biosynthetic genes *CPD* and *DWF4* or BR inactivation gene *BAS1* in 4-week-old plants. *ACT2* served as an internal control. (**C**) Root growth from 7-day-old seedlings grown on 1/2 MS medium with or without 100 nM BL. Scale bar, 1.5 cm. (**D**) Measurements of root length displayed in (C), n = 10 seedlings.The statistical analysis was performed to compare the root length treated with versus without BL samples, $**P < 0.001$ as two-way ANOVA with Sidak's multiple comparison test.

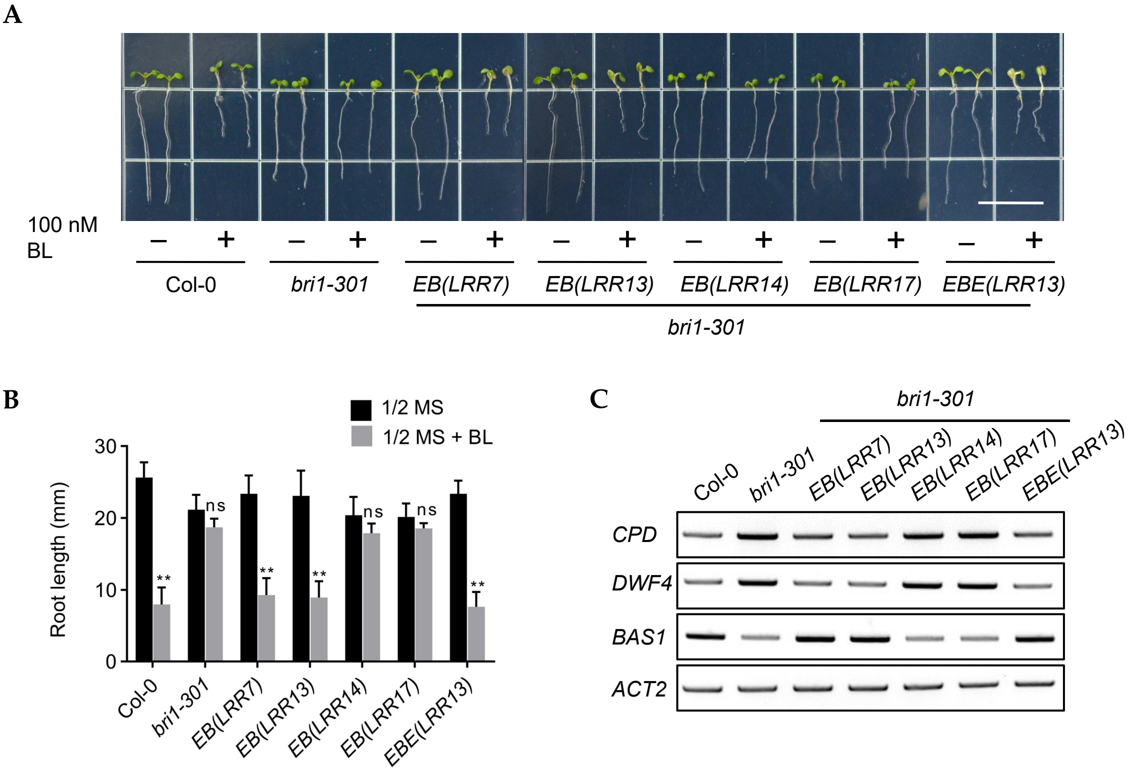

Figure S2. EB(LRR7), EB(LRR13) and EBE(LRR14) could restore *bri1-301*. (**A**) Root growth from 7-day-old seedlings of *EB(LRR7)/bri1-301*, *EB(LRR13)/bri1-301*, *EB(LRR14)/bri1-301*, *EB(LRR17)/bri1-301* and *EBE(LRR13)/bri1-301* grown on 1/2 MS medium with or without 100 nM BL. Scale bar, 1.5 cm. (**B**) Measurements of root length displayed in (A), n = 10 seedlings.The statistical analysis was performed to compare the root length treated with versus without BL, $^{**}P < 0.001$ as two-way ANOVA with Sidak's multiple comparison test. (**C**) Semi-quantitative PCR analysis of BR biosynthetic genes *CPD* and *DWF4* or BR inactivation gene *BAS1* in 4-week-old plants. *ACT2* served as an internal control.

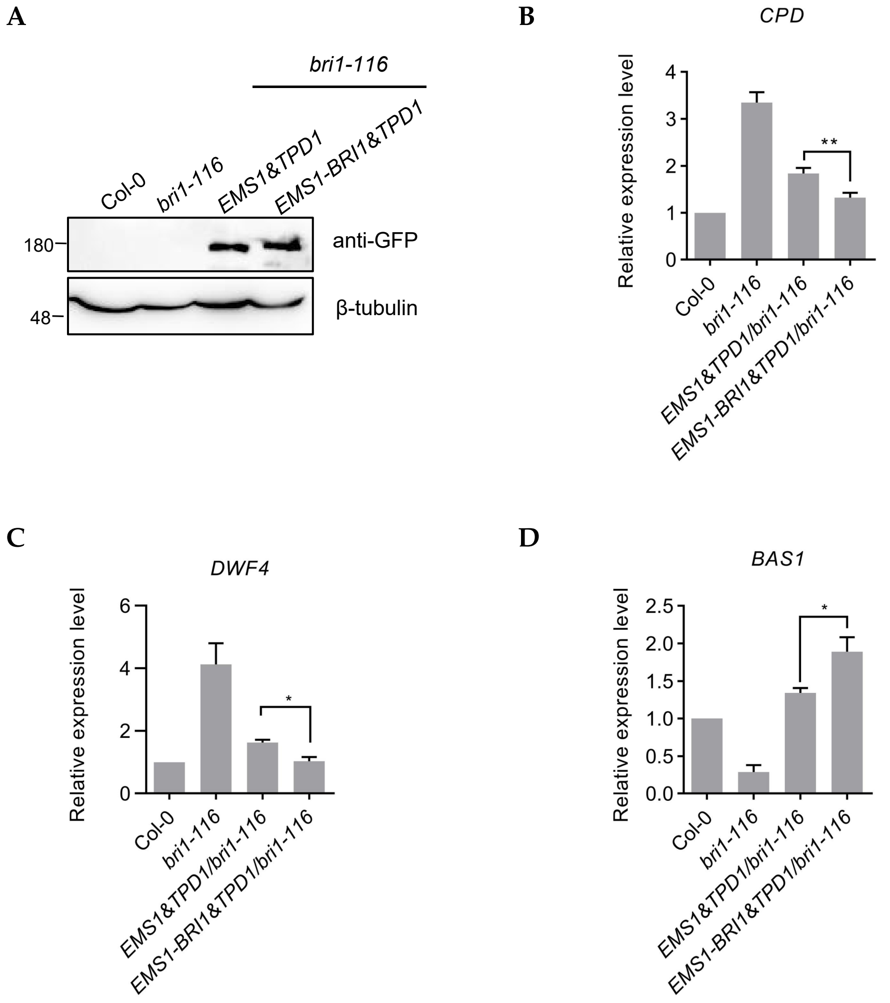

Figure S3. BR-related gene expression in *EMS1&TPD1/bri1-116* and *EMS1-BRI1&TPD1/bri1-116* transgenic plants. (**A**) The protein expression levels of *EMS1&TPD1/bri1-116* and *EMS1-BRI1&TPD1/bri1-116.* β-tubulin served as a loading control. (**B**-**D**) Quantitative real-time PCR analysis of BR biosynthetic genes *CPD* and *DWF4* or BR inactivation gene *BAS1* in 4-week-old plants. n = 4 biological replicates. **$P < 0.001$ (t-test).

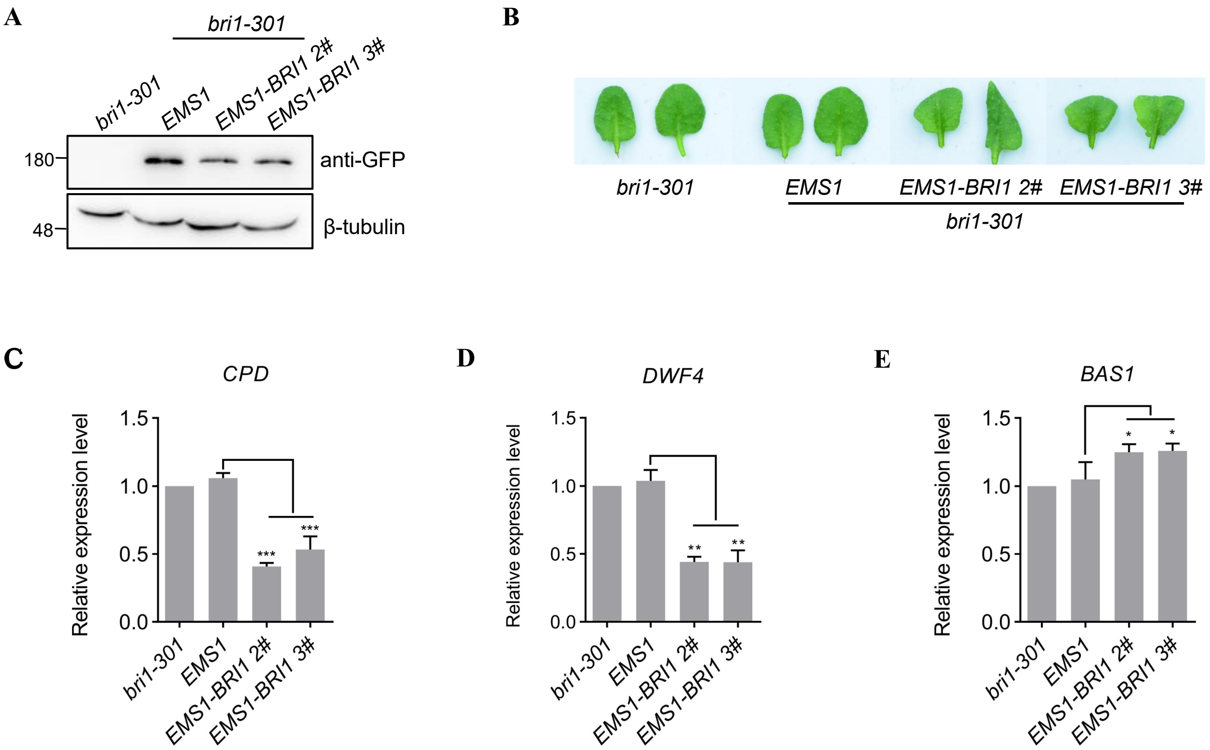

Figure S4. The phenotypes of *EMS1/bri1-301* and *EMS1-BRI1/bri1-301* transgenic plants. (**A**) The protein expression levels of *EMS1/bri1-301* and *EMS1-BRI1/bri1-301.* β-tubulin served as a loading control. (**B**) Rosette leaf phenotype of *bri1-301, EMS1/bri1-301* and *EMS1-BRI1/bri1-301.* (**C**-**E**) Quantitative real-time PCR analysis of BR biosynthetic genes *CPD* and *DWF4* or BR inactivation gene *BAS1* in 4-week-old plants. n = 3 biological replicates. *$P < 0.01$,**$P < 0.001$ (t-test).

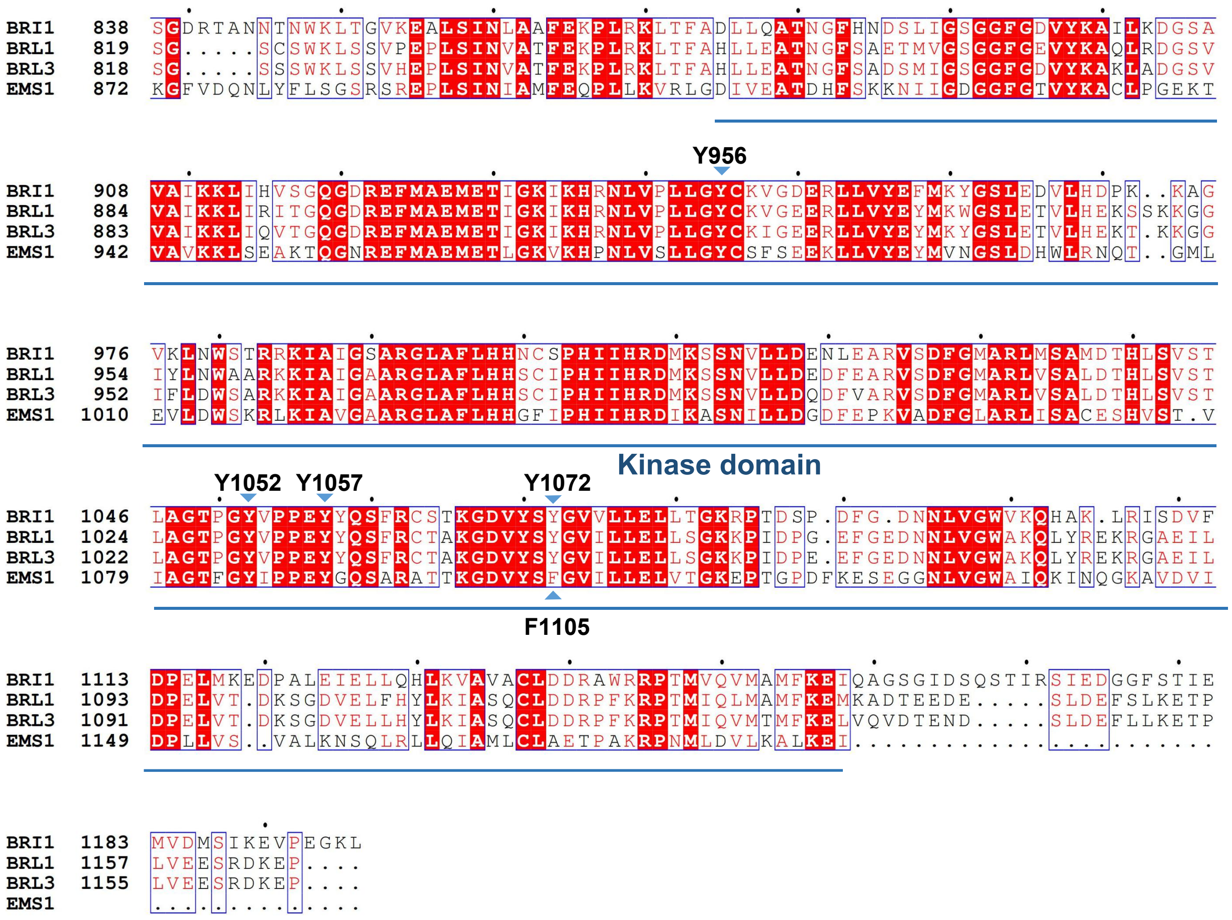

Figure S5. Sequence alignments of BRI1, BRL1, BRL3, and EMS1 kinase domain. Conserved residues are highlighted with red. The BRI1 Y956, BRI1 Y1052, BRI1 Y1057, BRI1 Y1072 and EMS1 F1105 are marked with arrowhead.

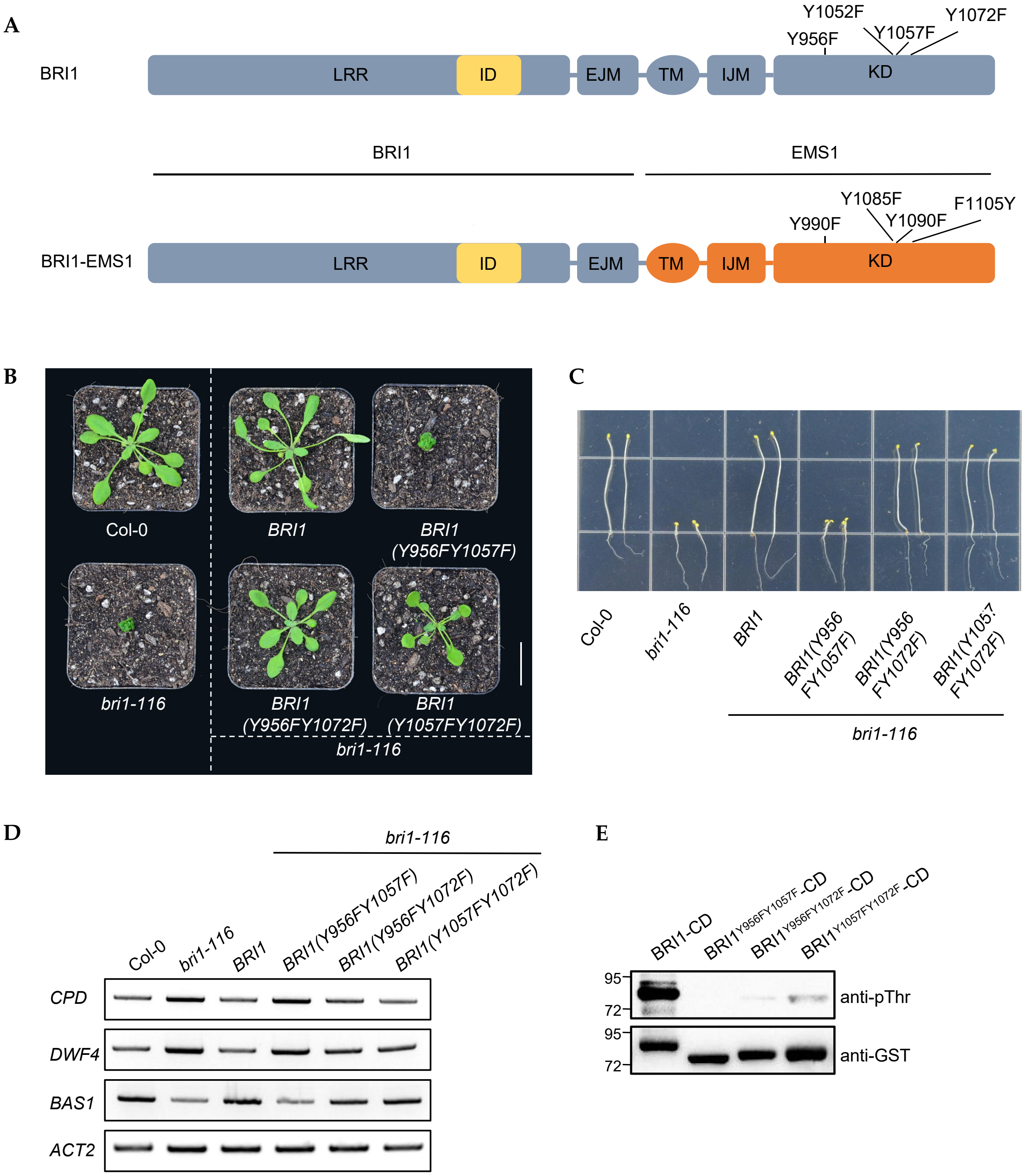

Figure S6. BRI1 (Y956F Y1057F) double sites mutant cannot rescue the phenotype of *bri1-116*. (**A**) Site-directed mutation strategy of BRI1 and EMS1 (**B**) Phenotypes of 4-week-old transgenic plants with double sites mutations in *bri1-116* mutant, Scale bar, 2.0 cm. (**C**) Hypocotyl phenotypes of 7-day-old transgenic plants in the dark. (**D**) Semi-quantitative PCR analysis of BR biosynthetic genes *CPD* and *DWF4* or BR inactivation gene *BAS1* in 4-week-old plants. *ACT2* served as an internal control. (**E**) The kinase activity of recombinant proteins BRI1-CD, BRI1^{Y956FY1057F}-CD, BRI1^{Y956FY1072F}-CD and BRI1^{Y1057FY1072F}-CD. Phosphorylation changes analyzed by pThr/Tyr antibody. GST served as the loading control.

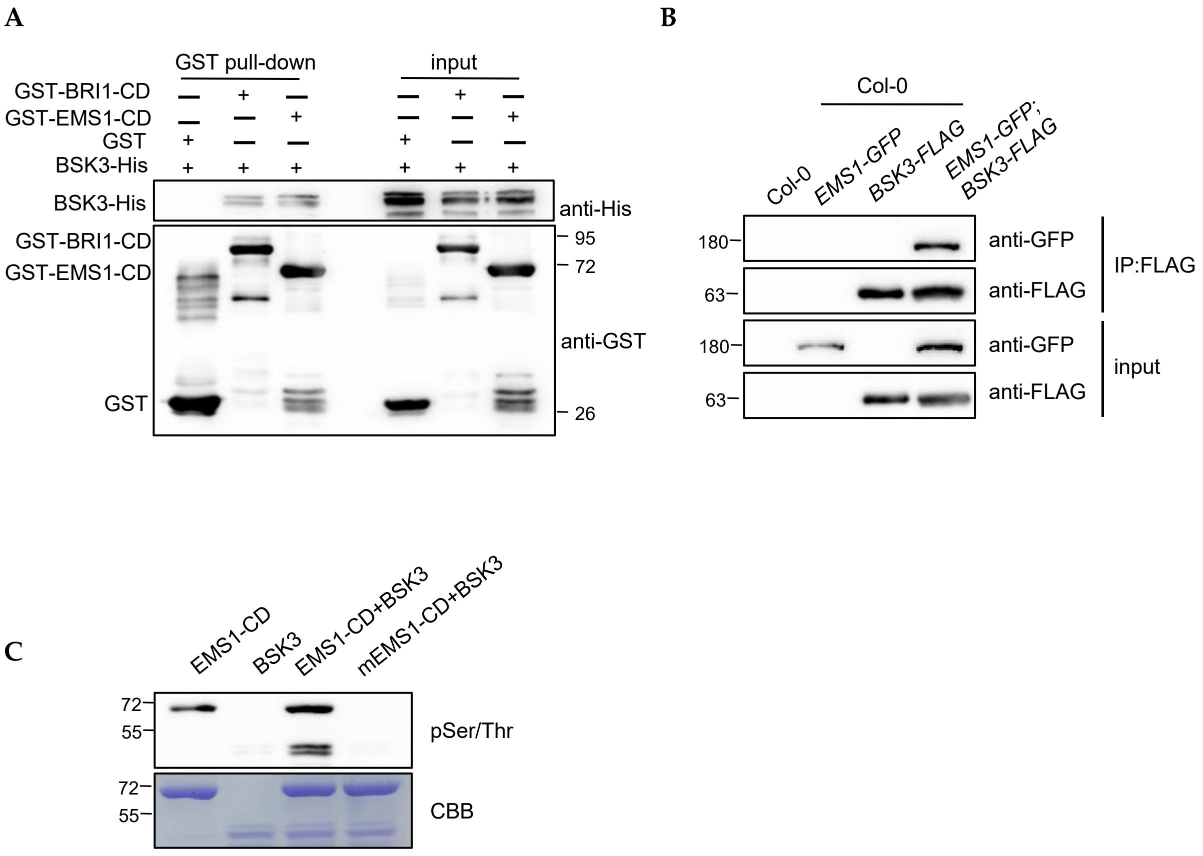

Figure S7. BSK3 is the substrate of EMS1. (**A**) Pull-down assays showed BRI1 and EMS1 could interact with BSK3, respectively. Immunoblot analysis is detected with His antibody and GST antibody. (**B**) EMS1 interacts with BSK3 in vivo. Total protein was extracted from 1-week-old Col-0, EMS1-GFP/Col-0, BSK3-FLAG/Col-0 and EMS1-GFP; BSK3-FLAG/Col-0 seedlings, immunoprecipitated with anti-FLAG M2 affinity gel, and then analyzed by GFP antibody and FLAG antibody. (**C**) The kinase assays were performed using EMS1-CD-GST, mEMS1-CD-GST and BSK3-His. Phosphorylation analyzed by pSer/Thr. The gel was stained with CBB.

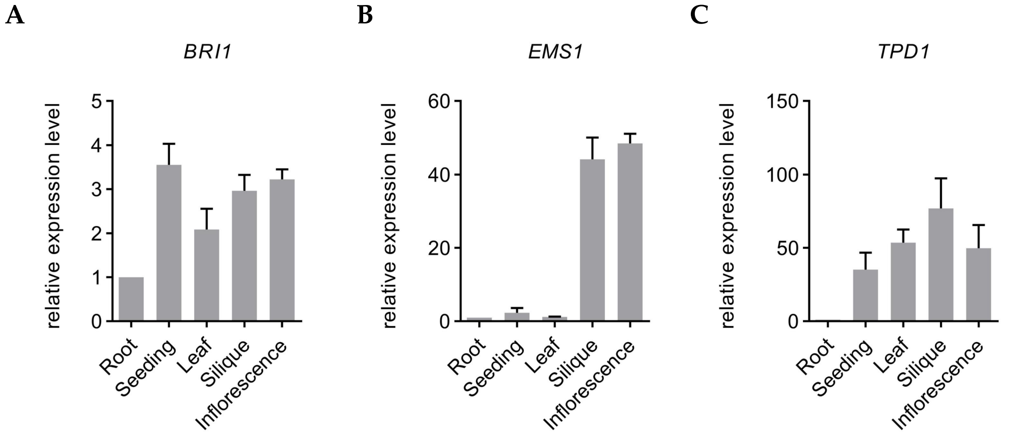

Figure S8. The expression of *BRI1*, *EMS1* and *TPD1* in different tissues. (**A**-**C**) Quantitative real-time PCR analysis of *BRI1*, *EMS1* and *TPD1* in root, seeding, leaf, silique and inflorescence.

Table S1. Primers used in this study.

Used for gene cloning	
BRI1-F-Kpn Ⅰ	GCGGTACCATGAAGACTTTTTCAAGCTTCTTTC
BRI1-R-Sal Ⅰ	GCGGTCGACTAATTTTCCTTCAGGAACTTCTT
EMS1-F-Kpn Ⅰ	GGTACCATGGCGTTTCTTACCGCATTGTTC
EMS1-R-Sal Ⅰ	GTCGACTCCTATCTCCTTAAGAGCCTTCAAC
BRI1-EMS1-KD-F	CACGTTTGCGGATATTGTCGAGGCTAC
BRI1-EMS1-KD-R	GTAGCCTCGACAATATCCGCAAACGTG
BRI1-EMS1-TM-F	GAAGGAGACCAGCGTGGGGGATTGCAG
BRI1-EMS1-TM-R	CTGCAATCCCCCACGCTGGTCTCCTTC
BRI1-EMS1-EJM-F	CTAAGTTCTTGAACAATAAAGAGTTGTGTG
BRI1-EMS1-EJM-F	CACACAACTCTTTATTGTTCAAGAACTTAG
BRI1-EMS1-ID-F	CGATATCTCTGGTGAAATACCTTCAGAAC
BRI1-EMS1-ID-R	GTTCTGAAGGTATTTCACCAGAGATATCG
EMS1(LRR7)-BRI1-F	GGAGAGTTGCATAATCTGCAACATCTTGAC
EMS1(LRR7)-BRI1-R	GTCAAGATGTTGCAGATTATGCAACTCTCC
EMS1(LRR13)-BRI1-F	CATTGATGGCACTTGATCTCAGCTC
EMS1(LRR13)-BRI1-R	GAGCTGAGATCAAGTGCCATCAATG
EMS1(LRR14)-BRI1-F	GAAATCAACGAACCTGCAGGAGCTTTAC
EMS1(LRR14)-BRI1-R	GTAAAGCTCCTGCAGGTTCGTTGATTTC
EMS1(LRR17)-BRI1-F	GTGATTGTACTTCTTTAGAGACTCTG
EMS1(LRR17)-BRI1-R	CAGAGTCTCTAAAGAAGTACAATCAC
EMS1-BRI1-F	GTTGAGGAGTGCTTCCCTTGCTGGTAG
EMS1-BRI1-R	CTACCAGCAAGGGAAGCACTCCTCAAC
TPD1-F-Kpn Ⅰ	GGTACCATGAACCGACGGCGACTTTTG
TPD-R-Sal Ⅰ	GTCGACCTAAGCACATGTCACGAAGGC
BSK1-Kpn Ⅰ -F	GGTACCATGGGTTGTTGTCAATCCTTG
BSK1-BamH Ⅰ -F	GGATCCATGGGTTGTTGTCAATCCTTG
BSK1-Sal Ⅰ -R	GTCGACAGATCCTCTGCCGCCTCG
BSK3-Kpn Ⅰ -F	GGTACCATGGGAGGTCAATGCTCTAGC
BSK3-BamH Ⅰ -F	GGATCCATGGGAGGTCAATGCTCTAGC
BSK3-Sal Ⅰ -R	GTCGACCTTCACTCGGGGAACTCCATTC
BSK3-R-Xma Ⅰ	CCCGGGCTTCACTCGGGGAACTCC
BRI1-CD-F-BamH Ⅰ	GGATCCAGAGAGATGAGGAAGAGACGG
BRI1-CD-R-Sal Ⅰ	GTCGACTCATAATTTTCCTTCAGGAACTTC
BRI1-Y956F-F	GCTTCTTGTGTTTGAGTTTATGAAG
BRI1-Y956F-R	CTTCATAAACTCAAACACAAGAAGC
BRI1-Y1052F-F	GTACACCGGGTTTCGTTCCTCCAGAG
BRI1-Y1052F-R	CTCTGGAGGAACGAAACCCGGTGTAC
BRI1-Y1057F-F	GTTCCTCCAGAGTTTTACCAAAGTTTCAG
BRI1-Y1057F-R	CTGAAACTTTGGTAAAACTCTGGAGGAAC

BRI1-Y1072F-F	GACGTTTATAGTTTCGGTGTGGTC
BRI1-Y1072F-R	GACCACACCGAAACTATAAACGTC
BRI1-E1078K-F	GTGTGGTCTTACTCAAGCTACTCACGG
BRI1-E1078K-R	CCGTGAGTAGCTTGAGTAAGACCACAC
EMS1-CD-F-BamH Ⅰ	GGATCCCGCAGATGGGCTATGACAAAG
EMS1-CD-R-Sal Ⅰ	GTCGACTCATATCTCCTTAAGAGCCTTCAAC
EMS1-Y990F-F	GCTTCTGGTATTTGAGTATATGGTAAATG
EMS1-Y990F-R	CATTTACCATATACTCAAATACCAGAAGC
EMS1-Y1085F-F	GACTTTCGGGTTTATCCCACCGGAGTATG
EMS1-Y1085F-R	CATACTCCGGTGGGATAAACCCGAAAGTC
EMS1-Y1090F-F	CACCGGAGTTTGGTCAGAGTGCCCGAG
EMS1-Y1090F-R	CTCGGGCACTCTGACCAAACTCCGGTG
EMS1-F1105Y-F	GATGTCTATAGCTATGGCGTGATACTATTG
EMS1-F1105Y-R	GCGGTACCATGAAGACTTTTTCAAGCTTCTTTC
EMS1-E1111K-F	GTGATACTATTGAAGCTTGTCACAGG
EMS1-E1111K-R	CCTGTGACAAGCTTCAATAGTATCAC
ProBRI1-F-Sal Ⅰ	GTCGACTAACATCAATGGCTAAG
ProAtBRI1-R-Xma Ⅰ	CCCGGGTTCTCAAGAGTTTGTGAGAGAG
ProAtEMS1-F- Sal Ⅰ	GTCGACCAGAGAGAACCAATGCAAC
ProAtEMS1-R-Xma Ⅰ	CCCGGGGTTCTTTTAGAGAAGGAGG
ProAtTPD1-F-Sal Ⅰ	GTCGACACATAGAGCTTGCATATATTTGG
ProAtTPD1-R-Xma Ⅰ	CCCGGGGTGCGTAGACGTCGAAGAAC
Used for RT and qRT-PCR	
ACT2-RT-F	ACTCTCCCGCTATGTATGTCG
ACT2-RT-R	TGGACCTGCCTCATCATACTC
CPD-RT-F	GTTCTTATCCTGCTTCCATTTG
CPD-RT-R	AGCCACTCGTAGCGTCTCATT
DWF4-RT-F	CGAAGGAAGGCTCTTTGAATG
DWF4-RT-R	CTTCAACGGCTTTAGGGCAA
BAS1-RT-F	GTTCAGGACATTGTGGAGGAG
BAS1-RT-R	GGATAAAGCAACATAAGGACG
ACT2-qRT-R	AGAAACCCTCGTAGATTGGC
CPD-qRT-F	GCAATGACGGATGTTGAGAT
CPD-qRT-R	CAAGGGTTGAAAGTGCGAGC
DWF4-qRT-F	AACAGACGATGATCTTTTGGG
BAS1-qRT-F	GCCAAATTGACACTCGCTGTAA
BAS1-qRT-R	GACGGTAGGTGCATGCTGATAA
EMS1-qRT-F	ATAGCAATGTTCGAGCAGC
EMS1-qRT-R	CAAAACCACCGTCTCCTAT
BRI1-qRT-F	GTCTATGGAGGTCACACTTCGC
BRI1-qRT-R	TTGAGAATAAACAGATAAGGCAT

TPD1-qRT-F	TCGTCTCCGTTGAAGCCTC
TPD1-qRT-R	GCAGCATTTTCCGATGTGAT
Use for genotyping	
bri1-116-F	TGGCGAGTTACCGATGGATACG
bri1-116-R	CTCTTAGATCACCTACCTCATCAGG
bri1-116-T-R	GACCCAAGGAAAATCGGACTGACC
Ds-LB	CGTTCCGTTTTCGTTTTTTACC
ems1-gt-F	AACAAACCCCGTCAGCTTTA
ems1-gt-R	ACCGGAGAAGTGGTTGTCAC
EMS1-F1-LP-1950	GCCCATTCCCTTCAGTAAAC
EMS1-R1-RP-213	GGAGAGCGAGTTGACTCGTC
